# A Global Public Database of Disease Vector and Reservoir Distributions

**DOI:** 10.1371/journal.pntd.0000378

**Published:** 2009-03-31

**Authors:** Alexander Moffett, Stavana Strutz, Nelson Guda, Camila González, Maria Cristina Ferro, Víctor Sánchez-Cordero, Sahotra Sarkar

**Affiliations:** 1 Biodiversity and Biocultural Conservation Laboratory, Section of Integrative Biology, University of Texas at Austin, Austin, Texas, United States of America; 2 Environmental Science Institute, University of Texas at Austin, Austin, Texas, United States of America; 3 Laboratorio de Sistemas de Información Geográfica, Departamento de Zoología, Instituto de Biología, Facultad de Medicina, Doctorado en Ciencias Biomédicas, Coyoacán, México; 4 Instituto Nacional de Salud Bogotá, Bogotá, Colombia; Swiss Tropical Institute, Switzerland

## Introduction

The Disease Vector Database is a global, free, public, Web-accessible resource presenting data on the geographical distribution of infectious disease vectors and reservoirs. At present, the Database contains records for dengue and malaria vectors and Chagas disease and leishmaniasis vectors and reservoirs. Future versions of the Database will include parasite data. These data can be used for a variety of purposes including the construction of ecological niche models and disease risk maps. The Database permits downloading data in formats designed to facilitate such use, for instance, as input files for popular niche modeling software packages such as Maxent and GARP.

## Background

Vector-borne infectious diseases adversely affect the health of large numbers of people globally. Effective response to these diseases requires representations of risk, which often take the form of risk maps [1]–[3], with geographic estimates of risk based on distributions of parasite, vector, and reservoir species in addition to human population, social organization, and behavior. The feasibility of constructing risk maps has increased substantially in recent years due to the widespread availability of digital environmental layers, geographic information system (GIS) platforms, and advances in ecological niche modeling, which makes it possible to produce maps even with very few parasite, reservoir, and vector presence records [3]. Niche models have been used to predict the geographic distributions of Chagas disease and leishmaniasis vectors and reservoirs and dengue and malaria vectors 3–5.

The construction of risk maps and other forms of spatial risk assessment require the availability of georeferenced parasite, vector, and reservoir records. Several groups have suggested the creation of public repositories of such spatial data [6],[7]. We have created the Disease Vector Database (http://www.diseasevectors.org), a global, free, publicly accessible Web-based database for disease vector and reservoir data. Future versions of the Database will include parasite data. At present, the Database has occurrence records for vector and reservoir species for Chagas disease and leishmaniasis, and vector species for dengue and malaria (Table 1). Researchers from around the world may submit data for inclusion in the Database following a submission protocol similar to that of GenBank [8].

**Table 1:** Summary of the current contents of the Disease Vector Database.

Disease	Vectors			Reservoirs		
	Species	Records	Minimum/Maximum Records per Species	Species	Records	Minimum/Maximum Records per Species
Chagas disease	62	567	1/159	45	7,492	3/1,353
Dengue	3	720	45/569	—	—	—
Leishmaniasis	15	415	1/102	9	1,103	4/1,024
Malaria	93	5,400	1/1,265	—	—	—

## Existing Databases

There are some existing databases for disease vectors, mainly for malaria. However, compared to the effort being reported here, each of these databases has some limitations (though some provide useful additional data beyond the scope of the Database described here). In 1996, the Mapping Malaria Risk in Africa (MARA) collaboration (http://www.mara.org.za/) began to provide a repository of information on malaria-transmission intensities in Africa [9]. Though the focus of the collaboration was on parasite data rather than on vector data, it currently contains 2,535 georeferenced records of malaria vectors. However, the records cover only the six members of the *Anopheles gambiae* complex, and there is no information from continents other than Africa. Koum and colleagues (2005) [10] created a database of malaria vectors with georeferenced information sampled from 19 African locations. However, the focus was on establishing the proper ontology for such a database and not on public use. The Walter Reed Army Institute and the Smithsonian Institution's MosquitoMap Web site (http://www.mosquitomap.org/) contains distributional information on mosquito species.

## Database Coverage

Chagas disease, dengue, leishmaniasis, and malaria were selected for initial inclusion in our Database because of the epidemiologic importance of vector and reservoir control for the prevention of their transmission and the availability of data. For the Database, the vectors and reservoirs implicated in the four diseases were identified from reviews. There were three sources for the data. First, a number of the data points (for instance, ∼10% of the reservoir data and ∼75% of the leishmaniasis vector data) came from field records of collaborators collected over several decades. This will eventually be the most important source of new data. Second, the ISI Web of Knowledge and Google Scholar were searched using “distribution” along with the genus and name of each species. References from the publications identified on the basis of this search were also consulted for additional data. Third, records from public databases such as MANIS (for mammal reservoir data; http://manisnet.org/), MARA, and MosquitoMap were included.

A record is included in the Database only if it meets the following criteria: (a) it represents a confirmed or suspected disease vector or reservoir; (b) it includes the species of the vector or reservoir; (c) it can be georeferenced to at least the nearest arc-minute. When available, additional information is included for the following: abundance, collection method, collector contact information, country, date, location, provenance, region, and voucher location. Each record has a unique accession number; information is maintained on the dates of initial inclusion and last modification. Data on the origin of the information are also retained. Records referenced only by names of study sites or by points on published maps were included only if they could be adequately georeferenced. The spatial data associated with these records may be less accurate than those for records with published coordinates: The Database records such uncertainties so that users can decide whether records are sufficiently precise for their needs. Table 2 discusses advantages and disadvantages of the Database.

**Table 2:** Advantages and disadvantages of the Disease Vector Database.

Advantages	Disadvantages
• Central source of data for a variety of diseases, with collaborators continually uploading new data.	• Data may appear to be more precise than they actually are, with limitations not being explicitly realized.
• Provides georeferenced data that can be used for ecological niche modeling and to produce risk maps.	• Data from different sources may have different levels of reliability with nothing to indicate the problem explicitly.
• Data can be easily visualized.	• This Database does not contain disease incidence records or any information on human factors of disease risk.

## Potential Uses

**Figure 1:**
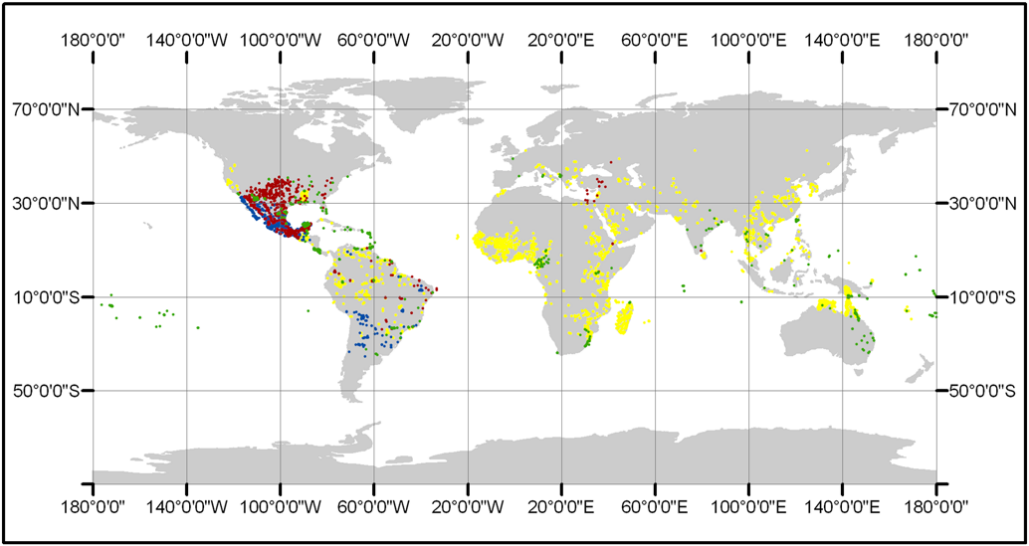
The distribution of vector occurrence data currently contained in the Disease Vector Database. Yellow, malaria vectors; green, dengue vectors; blue, Chagas reservoirs and vectors; red, leishmaniasis reservoirs and vectors.

The Web site enables the submission of data from an unlimited number of collaborators, who are then listed on the Web page. Data from the Disease Vector Database can be used in a variety of ways to help us understand the geographic risk of disease. Restricting attention to publicly available data, maps of occurrence records from the Database can be used to identify areas that need attention. For instance, the neotropics south of México in Central America have not been adequately sampled for Chagas disease vectors and reservoirs (see Figure 1). Data on potential leishmaniasis reservoirs are lacking from areas outside North America; data on vectors are incomplete except in Latin America. As additional existing data are incorporated into the Database, the geographic limitations will become less severe. Nevertheless, it is likely that there will remain significant areas with no data because they have not been surveyed. Compared to malaria and dengue, strikingly little data are publicly available for Chagas disease and leishmaniasis.

The other most obvious use of such data is for ecological niche modeling. For malaria, this Database has already been used to predict continent-wide distribution maps for ten malaria vector species in Africa, with these maps then being used to produce relative risk maps [3]. The long-term goal of the Disease Vector Database is to include many more diseases. Success of the project will depend on active collaboration of researchers from around the world. The fact that this Database could be constructed already shows the extent to which free datasharing is becoming the norm rather than the exception in epidemiology and ecology. Success in extending the Database will depend on a continuation of this trend, and this note is intended to encourage further collaboration.
